# Low-intensity pulsed ultrasound activated the anti-tumor immunity by irradiating the spleen of mice in 4 T-1 breast cancer

**DOI:** 10.1007/s00262-023-03613-1

**Published:** 2024-02-13

**Authors:** Yi Xia, Meijie Yang, Xinfang Xiao, Wentao Tang, Juan Deng, Liu Wu, Haopeng Xu, Yilin Tang, Wenzhi Chen, Yan Wang

**Affiliations:** 1 State Key Laboratory of Ultrasound in Medicine and Engineering, College of Biomedical Engineering, Chongqing Medical University, 1 Yixueyuan Rd, Yuzhong District, Chongqing, 400016 China; 2 College of Medical Informatics, Chongqing Medical University, Chongqing, China

**Keywords:** Low-intensity pulsed ultrasound, Immune activation, Anti-tumor immunity, Breast cancer

## Abstract

Tumor immunotherapy is booming around the world. However, strategies to activate the immune system and alleviate the immunosuppression still need to be refined. Here, we demonstrate for the first time that low-intensity pulsed ultrasound (LIPUS, spatial average time average intensity (*I*_sata_) is 200 mW/cm^2^, frequency is 0.3 MHz, repetition frequency is 1 kHz, and duty cycle is 20%) triggers the immune system and further reverses the immunosuppressive state in the mouse models of breast cancer by irradiating the spleen of mice. LIPUS inhibited tumor growth and extended survival in mice with 4 T-1 tumors. Further studies had previously shown that LIPUS enhanced the activation of CD4^+^ and CD8^+^ T cells in the spleen and led to significant changes in cytokines, as well as induced upregulation of mRNA levels involved in multiple immune regulatory pathways in the spleen. In addition, LIPUS promoted tumor-infiltrating lymphocyte accumulation and CD8^+^ T cell activation and improved the dynamics of cytokines/chemokines in the tumor microenvironment, resulting in a reversal of the immunosuppressive state of the tumor microenvironment. These results suggest a novel approach to activate the immune response by irradiating the spleen with LIPUS.

**Supplementary Information:**

The online version contains supplementary material available at 10.1007/s00262-023-03613-1.

## Introduction

Immunotherapies are designed to mobilize endogenous immune mechanisms to powerfully target and fight cancer cells by manipulating their own immune system, while preserving the integrity of normal or healthy cells [1, 2]. Since immunity plays such a key role in the progression of tumors, immunotherapy has made tremendous progress as the fourth major tumor treatment modality after surgery, chemotherapy and radiotherapy [3]. Stimulation of the immune system has shown some promise in the treatment of tumors as well as immune activation may lead to the destruction and clearance of tumor cells or protein aggregates [4]. Immunotherapies such as cancer vaccination [5], cytotoxic T lymphocyte-associated antigen 4, checkpoint inhibitor immunotherapy [6] or chimeric antigen receptor T-cell immunotherapy [7] have been shown to mobilize the systemic immune system to fight tumors. However, the therapeutic effect of these immunotherapies to activate the immune system to entirely suppress local tumors remains limited [8, 9]. Meanwhile the tumor immunosuppressive microenvironment significantly diminishes the effective anti-tumor immune response [10]. The recruitment of various cells such as myeloid-derived suppressor cells, regulatory T cells (Tregs), tolerogenic dendritic cells (tolDCs) and tumor-associated macrophages in the tumor microenvironment puts it in an immunosuppressed state [11–13], which is responsible for tumor evasion of immune surveillance and leads to tumor growth and metastasis [14]. Therefore, activating the immune system and improving the immunosuppressive state of the tumor microenvironment in order to induce an effective anti-tumor immune response is essential for inhibiting tumor growth.

Low-intensity pulsed ultrasound (LIPUS) is a current and safe noninvasive physical therapy. ​Targeting the spleen with noninvasive ultrasound has been reported to activate anti-inflammatory pathways and alter the expression of genes involved in cytoskeletal regulation, thereby affecting lymphocyte polarization or migration [15]. In our preliminary study, LIPUS can treat cyclophosphamide-induced leukopenia in rabbits and significantly increase serum immunoglobulin (Ig) levels in rabbits [16]. In addition, LIPUS is effective on improving serum Ig levels and enhancing the immunity of the body after cyclophosphamide chemotherapy in a rat model of breast cancer [17].

Here we report on the ability of LIPUS irradiation of the spleen of mice to activate the immune system and further explore the mechanisms of activation. This suggests that LIPUS may offer a novel approach and an effective adjunct to immunotherapy in oncology.

## Materials and methods

### Animals

Six–eight-week-old female BALB/c mice were purchased from the Laboratory Animal Center of Chongqing Medical University (Production License No. SCXK 2021–0023, Chongqing, China), and all had body masses of 18 to 20 g. All mice were acclimatized in a specific pathogen-free laboratory animal facility for 1 week prior to the experiment. The mice were housed at a rearing temperature of (23 ± 1)°C and relative humidity of (40 ± 10%) for a 12-h light–dark cycle and fed with standard food and sterilized water ad libitum. All animal studies were conducted in accordance with the Guide for the Care and Use of Laboratory Animals and approved by the Ethics Committee of Chongqing Medical University (reference number 2022098).

### Cell culture and tumor inoculation

The 4 T-1 cell line was purchased from the Cell Bank of the Chinese Academy of Sciences. Cells were cultured in RPMI-1640 medium containing 10% fetal bovine serum and 100U/ml penicillin–streptomycin under 5% CO_2_ at 37 °C. To establish the tumor model, 1 × 10^6^ 4 T-1 cells were suspended in 100 μL phosphate-buffered saline (PBS) and injected subcutaneously into the right flank of BALB/c mice. When the tumor volume reached 200 mm^3^, tumor-bearing mice were used.

### LIPUS treatment

The LIPUS device was provided by Chongqing Ronghai Engineering Research Center of Ultrasound Medicine Co., Ltd, Chongqing, China. The spleen (it was located between shoulder joint and hip joint by dissecting the mice, so we chose these two positions to locate the spleen) of mice was irradiated by ultrasound for 20 min per day [18], and ultrasound energy reaches mouse spleen mediated by coupler. The ultrasound parameters were as follows: Spatial average time average intensity (*I*_sata_) was 200 mW/cm^2^, frequency was 0.3 MHz, repetition frequency was 1 kHz, and duty cycle was 20%. Both the Pre-Control group and the Control group received sham treatment (no probe energy output).

### Tumor growth and survival analysis

The following groups were set up according to the irradiation method and time. In the first modeling approach, normal mice were randomly divided into four groups (*n* = 5 per group), including: (1) Pre-LIPUS_7_, (2) Pre-LIPUS_14_, (3) Pre-LIPUS_21_ and (4) Pre-Control groups, and then 4 T-1 cells were injected subcutaneously after irradiating normal mice with LIPUS for 7, 14 and 21 days, respectively. Mice were euthanized on day 30 after 4 T-1 cell injection, tumors were excised and weighed, organs were collected.

In the second modeling approach, the tumor-bearing mice were divided into four groups (*n* = 18–20) randomly, including: (1) LIPUS_7_, (2) LIPUS_14_, (3) LIPUS_21_ and (4) Control groups and then irradiated with LIPUS for 7, 14 and 21 days, respectively. Mice were euthanized on day 30 after LIPUS irradiation, and the followed were the same as the aforementioned procedures. To observe the survival rate of mice after LIPUS irradiation, mouse survival was monitored for each group of tumor-bearing mice starting from the time of injection of 4 T-1 cells, while dead mice were excluded.

Tumor size was measured with digital calipers and the volume (mm^3^) was calculated according to the following formula: length × width^2^/2.

### Enzyme-linked immunosorbent assay (ELISA)

Blood, spleen and tumor tissues were isolated from mice. The levels of interleukin-2 (IL-2), interleukin-7 (IL-7), interleukin-10 (IL-10), interferon-gamma (IFN-γ), CXCchemokineligand-9 (CXCL9), CXCchemokineligand-10 (CXCL10) and transforming growth factor-β (TGF-β) were assessed using ELISA kits (Jingmei Biotechnology Co., Ltd., Jiangsu, China) according to the manufacturer’s recommended procedures.

### Flow cytometry (FCM)

Single cell suspensions are prepared labeled with antibodies. Adherent cells were excluded by FSC/A and FSC/H gating analysis. Cells were analyzed using CytoFLEX flow cytometry (Beckman Counter, USA). Data were analyzed by FlowJo version 10.8.1 (Ashland, OR, USA).

### Terminal deoxynucleotidyl transferase-mediated dUTP nick-end labeling (TUNEL)

Tumor tissues were isolated after 21 days of LIPUS irradiation, fixed in 4% paraformaldehyde at 4 °C and then dehydrated in 30% sucrose solution. Tumor tissue apoptosis was detected using the Cell Death Detection Kit, 4AF594 TUNEL Assay (4A Biotech Co., Ltd, Beijing, China) according to the manufacturer’s instructions. All the sections were imaged with fluorescence microscope (ECHO Revolve, USA).

### RNA-seq and bioinformatic data analysis

The spleen was removed after 21 days of LIPUS irradiation of the tumor-bearing mice, and all RNA sample processing and RNA-seq were performed by Shenzhen BGI Co., Ltd. GO analysis of DEGs was performed using DAVID (https://david.ncifcrf.gov/) with a corrected *P* value cutoff value of 0.05 to determine statistically significant enrichment. The gene set databases used in this analysis were downloaded from the Molecular Signature Database (http://software.broadinstitute.org/gsea/msigdb/index.jsp).

### Western blot

The spleen tissue was lysed in PIPA buffer containing 1% PMSF (Beyotime, Shanghai, China). After incubation with 5% nonfat milk in TBST (TBS containing 0.1% Tween-20) for 1 h at room temperature, the membranes were incubated with the anti-mouse IFN-γ antibody (Affinity Biosciences LTD., USA) overnight at 4 °C and visualized by enhanced chemiluminescence reagent (Bio-Rad Laboratories, Inc., USA). The protein was normalized to β-actin.

### Statistical analysis

Statistical analysis of all data was performed using GraphPad Prism 8.2.1 software (GraphPad Software Inc., San Diego, CA, USA). All continuous data were expressed by mean ± SEM. Two-factor (group × time) ANOVA test followed by Sidak’s multiple comparisons test was used to compare ELISA data between groups. The remaining continuous variables between two groups were tested using an unpaired two-sided Student’s *t* test or Mann–Whitney *U* test. One-way ANOVA followed by Turkey’s post hoc test was used to compare multiple groups. A log-rank (Mantel-Cox) test was used for survival data. Differences were considered statistically significant at *P < 0.05, **P < 0.01.

## Results

### LIPUS irradiation of normal mice spleen suppressed the growth of transplanted tumors

**Fig. 1:**
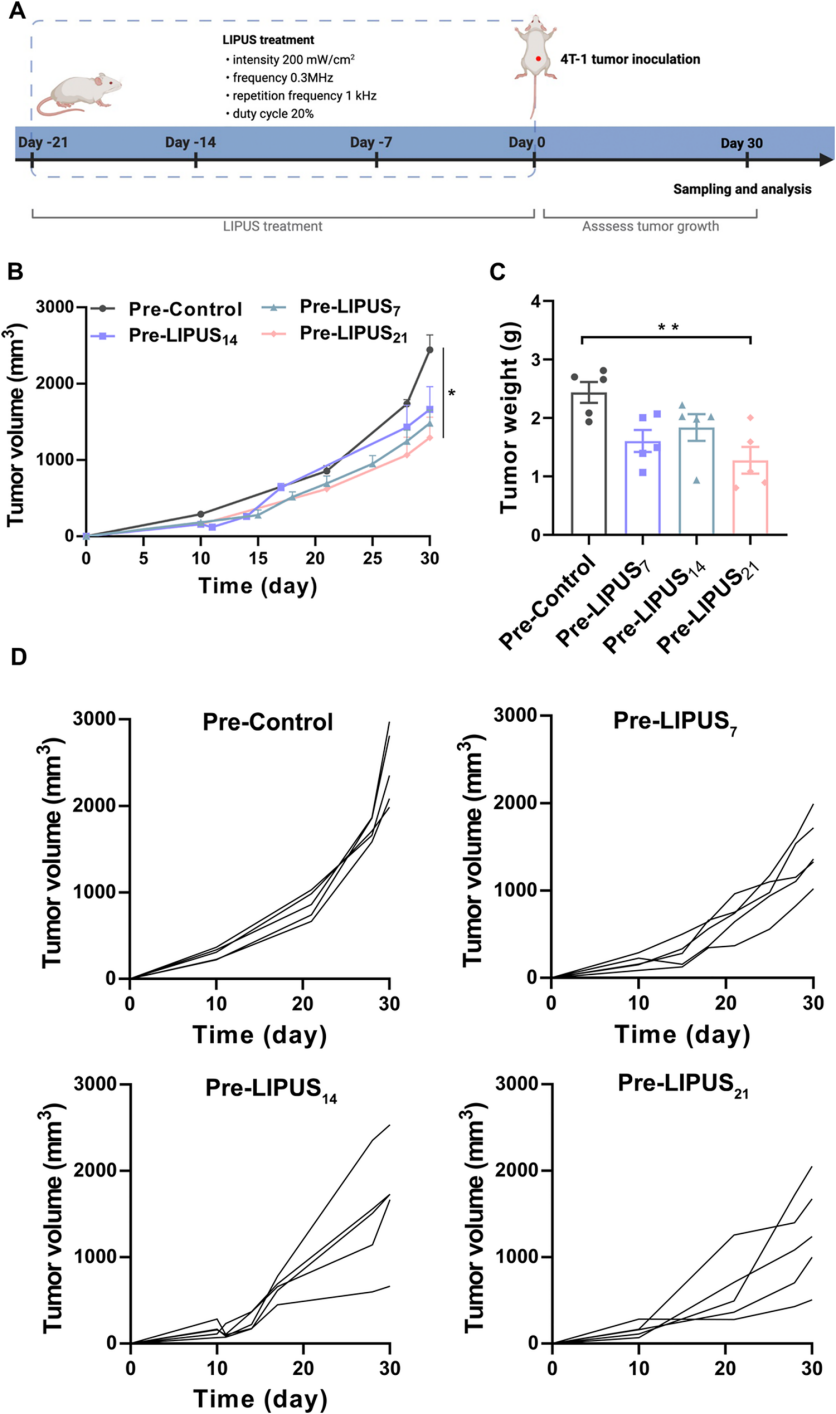
LIPUS irradiation of normal mice spleen suppressed the growth of tumors. **A** Experimental flow diagram. After LIPUS irradiation of normal mice spleen, 4 T-1 cells were inoculated in the right flank of mice (day 0) and mice were euthanized and tumors were excised on day 30 (*n* = 5 per group). **B** Tumor growth curves. **C** Tumor volume. **D** Individual tumor growth kinetics of the right tumors. Data are shown as mean ± SEM. *P < 0.05, **P < 0.01. *n.s.* no statistical significance

After the normal mice were irradiated, the 4 T-1 model was established for continuous observation, and tumors were excised on day 30 after inoculation (Fig. 1A). Tumor growth curves showed that LIPUS irradiation for 21 days significantly suppressed the tumor growth. The volume of the Pre-LIPUS_21_ group (1294 ± 267.4 mm^3^, *P* = 0.017) was significantly lower compared to the Pre-Control group (2443 ± 195.4 mm^3^) at day 30 (Fig. 1B-D). In addition, 4 T-1 tumors were excised and weighted, which mass in the Pre-LIPUS_21_ group (1.28 ± 0.23 g, *P* = 0.006) was also significantly lower than that in the Pre-Control group (2.44 ± 0.18 g) (Fig. 1C).

**Fig. 2:**
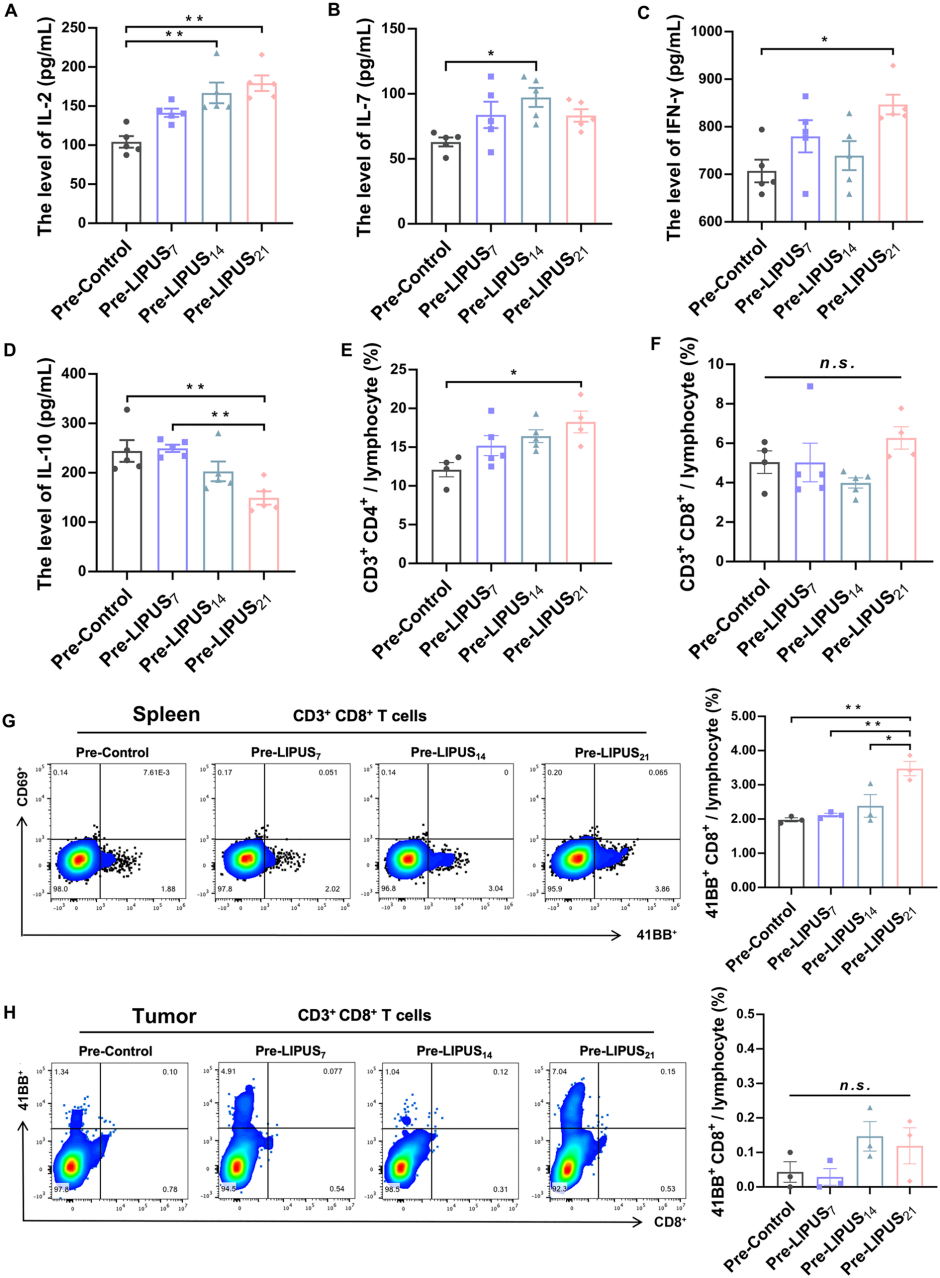
LIPUS-induced activation of CD8^+^ T cells and cytokine dynamics in the spleen of normal mice. **A**-**D** Plasma levels of IL-2, IL-7, IFN-γ and IL-10 cytokines in mice on day 30 (*n* = 5 per group). **E** Expression of CD3^+^CD4^+^ T cells in peripheral blood of mice on day 30 (n = 4 per group). **F** Expression of CD3^+^CD8^+^ T cells in peripheral blood of mice on day 30 (n = 4 per group). **G** Expression of 41BB^+^ and CD69^+^ gated on CD3^+^CD8^+^ T cells in the spleen (*n* = 3 per group). **H** Expression of 41BB^+^ gated on CD3^+^CD8^+^ T cells in tumors (*n* = 3 per group). Data are shown as mean ± SEM. *n.s.* no statistical significance, *P < 0.05, **P < 0.01

To confirm the immune response induced by LIPUS, the systemic immune response induced by LIPUS was first examined. The results showed significantly higher IL-2 levels in both the Pre-LIPUS_14_ group (*P* = 0.001) and the Pre-LIPUS_21_ group (*P* = 0.000) compared to the Pre-Control group (Fig. 2A). IL-7 levels were significantly higher in the Pre-LIPUS_14_ group (*P* = 0.014, Fig. 2B). It was also observed that IFN-γ levels were significantly higher in the Pre-LIPUS_21_ group than in the Pre-Control group (*P* = 0.012, Fig. 2C). The levels of IL-10 were significantly lower in the Pre-LIPUS_21_ group compared to both the Pre-Control group (*P* = 0.004) and the Pre-LIPUS_7_ group (*P* = 0.003) (Fig. 2D). The results showed that the proportion of CD4^+^ T cells in peripheral blood was significantly higher in the Pre-LIPUS_21_ group compared with the Pre-Control group (*P* = 0.013, Fig. 2E). But CD8^+^ T cells in peripheral blood did not alter significantly (all *P* > 0.05, Fig. 2F). These results suggested that LIPUS altered the secretion of these cytokines in the serum, as well as increasing the fraction of CD4^+^ T cells, effectively activating the body’s systemic immune response.

Next, we examined the functional status of CD4^+^ T cells and CD8^+^ T cells in the spleen of mice on day 30. The results showed no significant changes in the expression levels of Tregs (*P* = 0.298, Supplementary Fig. 1A) and CD4^+^ T cells activation marker CD69^+^ (*P* = 0.109, Supplementary Fig. 1B) between the groups. However, 41BB^+^ expression level was significantly increased in the Pre-LIPUS_21_ group compared to all other groups (all *P* < 0.05, Fig. 2G). We furthermore examined the functional status of infiltrated CD8^+^ T cells and also revealed no significant changes in the expression level of their activation marker 41BB^+^ (*P* = 0.168, Fig. 2H). These results suggested that activation of CD8^+^ T cells is expected to play a crucial role in triggering anti-tumor immunity.

### LIPUS irradiation of spleen in tumor-bearing mice suppressed tumor growth and prolonged survival time

**Fig. 3:**
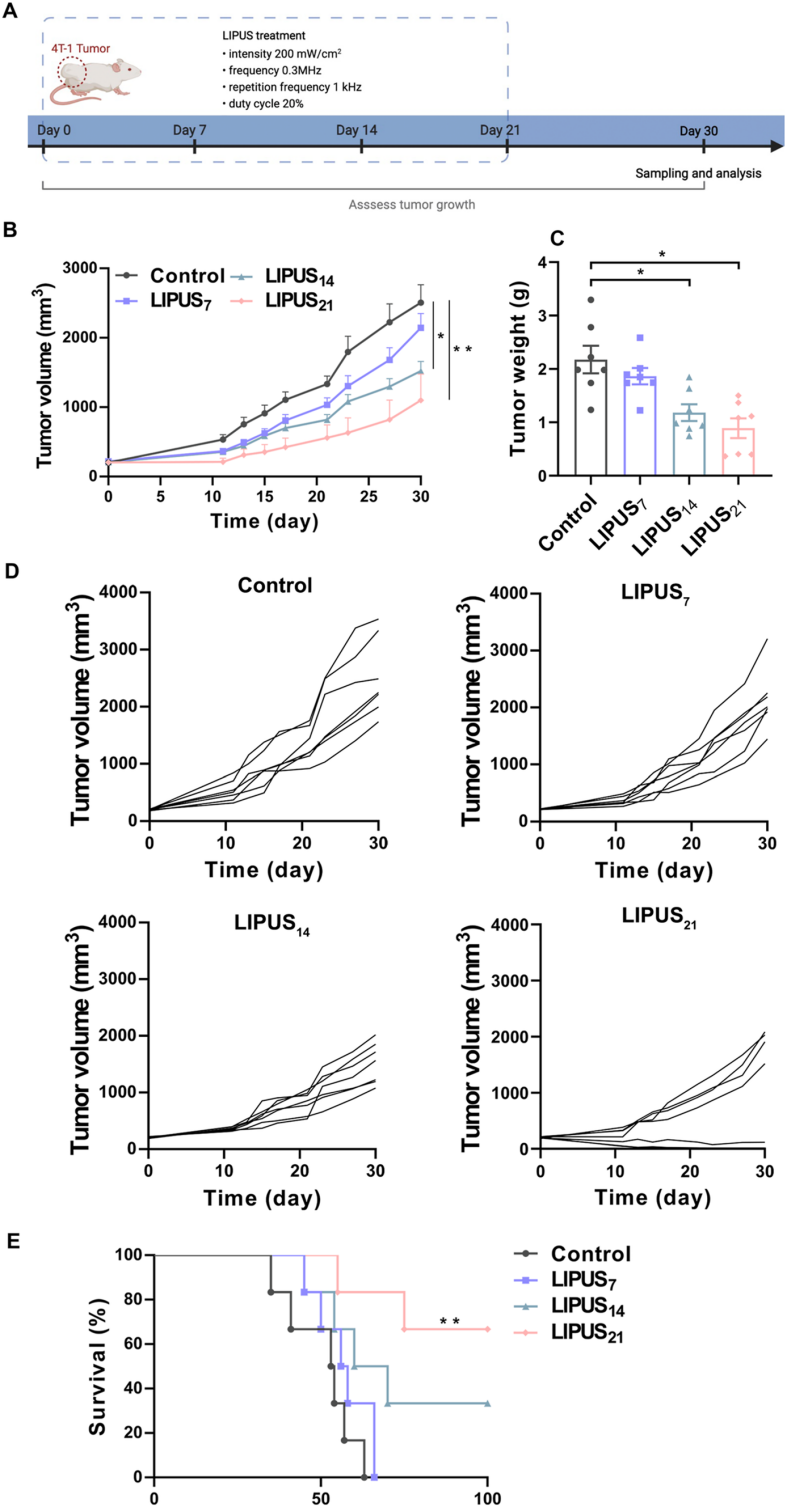
LIPUS irradiation of spleen in tumor-bearing mice suppressed tumor growth and prolonged survival time. **A** Experimental flow chart. 4 T-1 cells were inoculated in the right flank of mice (day 0) followed by LIPUS irradiation of the spleen, and the mice were euthanized and tumors excised on day 30 (*n* = 7 per group). **B** Tumor growth curves. **C** Tumor volume. **D** Individual tumor growth kinetics of the right tumors. **E** 100-day survival rate of mice inoculated with 4 T-1 cells (*n* = 6 per group). Data are shown as mean ± SEM. *P < 0.05, **P < 0.01

Our results indicating that LIPUS had immune activation function in normal mice; however, we found no significant infiltration of activated CD8^+^ T cells in the tumor microenvironment. In contrast, in our previous study, immunoglobulin (Ig) levels were significantly elevated after LIPUS irradiation in a damaged body such as the cyclophosphamide-induced myelosuppression model in rats with breast cancer [17]. So, we hypothesized that it is possible that normal mice have a limited capacity to respond to LIPUS immune activation. Therefore, the tumor growth suppression effect of LIPUS (after the modeling was completed, LIPUS was irradiated for 7, 14 and 21 days, respectively) was based on tumor-bearing mice. The tumors were excised and weighed on day 30 after irradiation (Fig. 3A). Another batch of mice was observed the survival of the mice for 100 days after inoculation with 4 T-1 tumor cells. Tumor growth curves showed that LIPUS significantly inhibited tumor growth, particularly in the LIPUS_21_ group. After 14 and 21 days of LIPUS irradiation, the volume at day 30 was significantly lower in the LIPUS_14_ group (1522 ± 361.4 mm^3^, *P* = 0.045) and in the LIPUS_21_ group (1099 ± 377.9 mm^3^, *P* = 0.004) than in the Control group (2570 ± 255.9 mm3) (Fig. 3B-D). Similarly, 4 T-1 tumors were excised and weighted, and the tumors mass was found to be significantly lower in the LIPUS_14_ group (1.18 ± 0.16 g, *P* = 0.006) and the LIPUS_21_ group (0.89 ± 0.18 g, *P* = 0.000) compared to the Control group (2.17 ± 0.26 g) (Fig. 3C). Tumor-bearing mice that received 21 days of LIPUS irradiation also benefited significantly in terms of survival, with 66.67 percent still alive at 100 days after inoculation. In contrast, all mice in the Control group died within 65 days of the inoculation (Fig. 3E). These results indicated that LIPUS successfully suppressed tumor growth as well as prolonged survival in the 4 T-1 tumor model.

### LIPUS irradiated spleen of tumor-bearing mice significantly enhanced anti-tumor immune effect

The activation of anti-tumor immune effects was further investigated on the basis that 21 days of LIPUS irradiation were able to significantly inhibit tumor growth and improve survival. In the peripheral blood, FCM results showed no significant changes in CD4^+^ and CD8^+^ T cells (Supplementary Fig. 2). IL-2 increased significantly after LIPUS irradiation (*P* = 0.012, Supplementary Fig. 3).

**Fig. 4:**
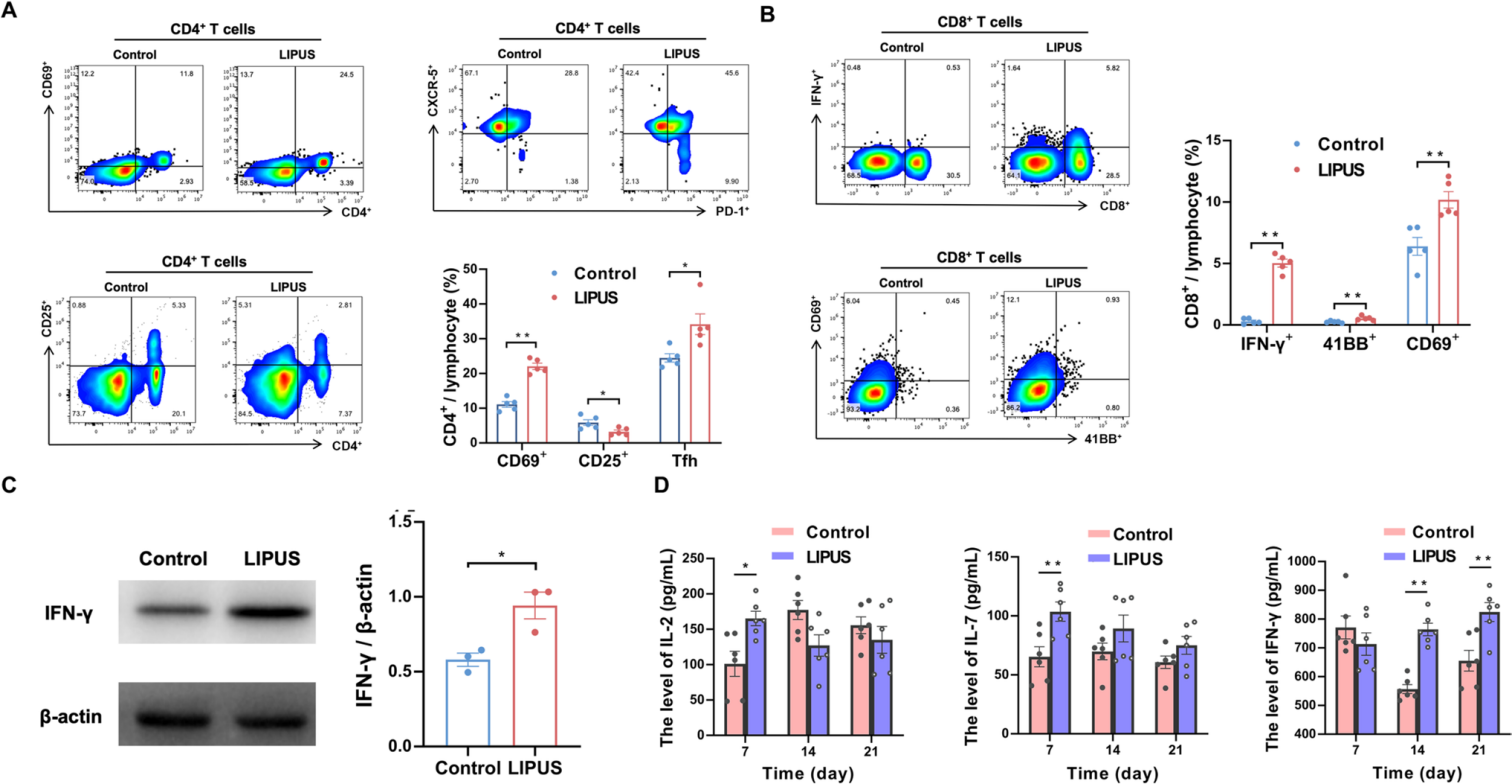
LIPUS-induced activation of CD4^+^ and CD8^+^ T cells in the spleen of tumor-bearing mice. **A** Expression of CD69^+^, CD25^+^ (Tregs) and CXCR5^+^ PD-1^+^ T follicular helper (Tfh) gated on CD4^+^ T cells in the spleen on day 21 (*n* = 5 per group). **B** Expression of IFN-γ^+^, 41BB^+^ and CD69^+^ gated on CD8^+^ T cells in the spleen (*n* = 5 per group). **C** Expression of IFN-γ in the spleen was analyzed by Western Blot (*n* = 3 per group). **D** IL-2, IL-7 and IFN-γ cytokine levels in the spleen (*n* = 6 per group). Data are shown as mean ± SEM. *P < 0.05, **P < 0.01

The cellular immune response induced by LIPUS was next assessed. FCM was used to analyze the functional status and cytokine expression of CD4^+^ and CD8^+^ T cells. The data showed that after 21 days of LIPUS irradiation, the expression of CD69^+^ (*P* = 0.000) was upregulated, while LIPUS decreased the proportion of Tregs (*P* = 0.023) and increased the expression of CXCR5^+^ PD-1^+^ follicular helper T cells (Tfh) in the spleen (*P* = 0.017. Figure 4A). We observed that LIPUS induced an increase in IFN-γ^+^ CD8^+^ T cells compared to the Control group and significantly increased the expression levels of CD69^+^ (*P* = 0.005) as well as 41BB^+^ (*P* = 0.008, Fig. 4B). Western Blot and ELISA results also reinforced that LIPUS significantly increased the expression level of splenic IFN-γ after 21 days of LIPUS irradiation (*P* = 0.022, *P* = 0.002, respectively, Fig. 4C-D).

**Fig. 5:**
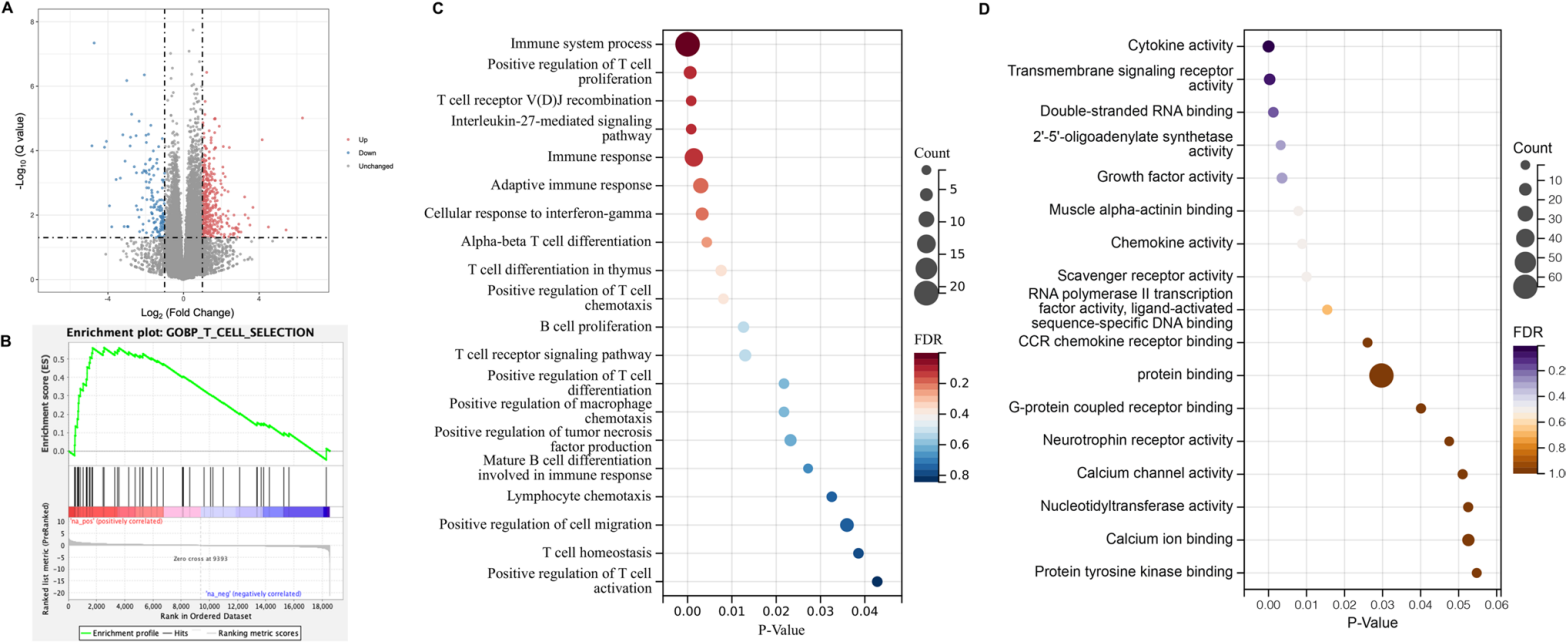
Transcriptome analysis of spleens from LIPUS irradiated tumor-bearing mice by RNA-seq. **A** Volcano plot showing the differentially expressed genes between the LIPUS group and the Control group (*n* = 5 per group). **B** GSEA analysis of T cell selection upregulated gene set in LIPUS group versus Control group. **C** Scatter plot for GO in terms of biological process (BP) enrichment results in the upregulated genes. **D** Scatter plot for GO in terms of molecular function (MF) enrichment results in the upregulated genes. The dot size indicates the number of DEGs contained in the GO terms, and the dot depth indicates the extent of rich factor enrichment

The transcriptome RNA-seq analysis on the spleen of tumor-bearing mice was performed. Compared to Control group, 1006 transcripts at a |log2(Fold Change)|> 1 and false discovery rate (FDR) < 0.05 were differentially expressed, of which 654 genes were upregulated and 352 genes were downregulated based on fragments per kilobase of exon per million mapped reads (FRKM) values (Fig. 5A). The gene ontology (GO) enrichment analysis using Gene Set Enrichment Analysis (GSEA) was applied, and data indicated that the LIPUS group had stronger T cell selection gene enrichment than the Control group, positively regulating the T cells for further maturation (Fig. 5B). The differentially expressed genes (DEGs) obtained using GO enrichment analysis were then analyzed. Among the upregulated genes, biological processes (BP) were assessed (Fig. 5C), molecular functions (MF) (Fig. 5D) and cellular localization (CC) (Supplementary Fig. 4) by GO enrichment. A series of terms related to the immune regulation was found, such as immune system process, positive regulation of T cell proliferation, T cell receptor V(D)J recombination, adaptive immune response, cellular response to IFN-γ, activation of T cells, positive regulation of macrophage chemotaxis. These results indicated that LIPUS has potent immune-activating properties and affects the spleen of tumor-bearing mice at the transcriptional level.

**Fig. 6:**
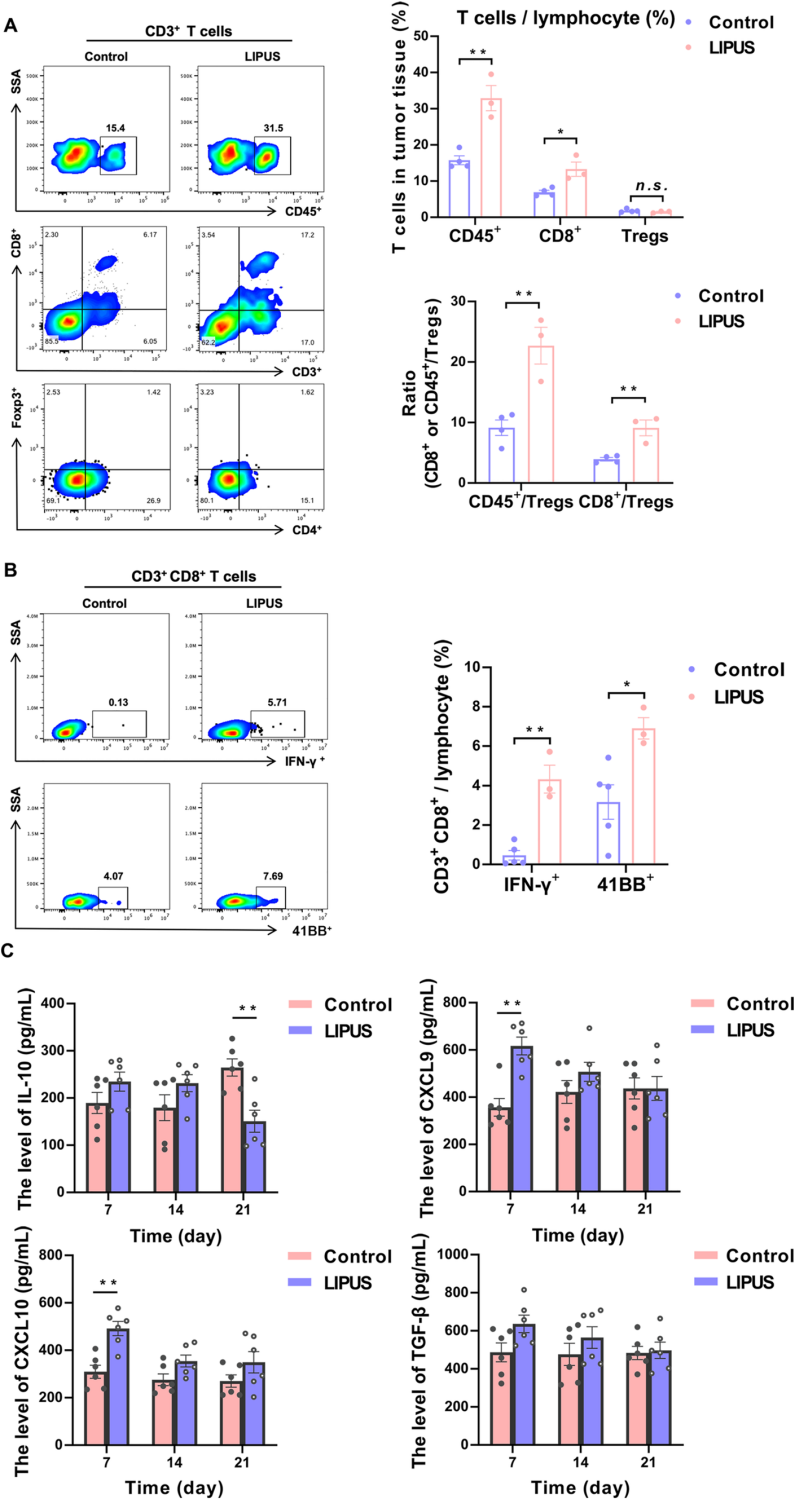
LIPUS increased lymphatic infiltration in tumors, thereby reversing the immunosuppressive state of the tumor microenvironment. **A** Tumor-infiltrating lymphocytes in tumor-bearing mice included CD45^+^ cells, CD8^+^ T cells and Tregs on day 21 (*n* = 3–5 per group). **B** Expression of IFN-γ^+^, 41BB^+^ gated on CD3^+^CD8^+^ T cells in tumors (*n* = 3–5 per group). **C** The levels of IL-10, CXCL9, CXCL10 and TGF-β cytokines in tumors (*n* = 6 per group). Data are shown as mean ± SEM. *n.s.* no statistical significance, *P < 0.05, **P < 0.01

Infiltration of leukocytes from the tumor is also essential for anti-tumor immune activation. Therefore, we again studied lymphocyte infiltration in the tumor of LIPUS irradiation for 21 days. FCM results showed that the proportion of leukocyte-specific antigen CD45^+^ was significantly higher in the LIPUS group compared to the Control group (*P* = 0.003). The proportion of CD8^+^ T cells was significantly higher (*P* = 0.016), while the proportion of Tregs (CD4^+^ Foxp3^+^ CD3^+^ T cells) did not modify significantly (*P* = 0.33). However, it was notable that the ratio of the number of CD45^+^ to Tregs in the LIPUS group (LIPUS: 22.70 ± 3.03, Control: 9.15 ± 1.28, *P* = 0.006) and the ratio of the number of CD8^+^ T cells to Tregs (LIPUS: 9.11 ± 1.29, Control: 3.93 ± 0.29, *P* = 0.006) were considerably higher than those of the Control group (Fig. 6A). This indicated that LIPUS contributed to an increase the homing of CD8^+^ T cells to the tumor tissue. The functional status of CD8^+^ T cells in the tumor microenvironment of mice was examined sequentially. FCM results showed that after 21 days of LIPUS irradiation, LIPUS upregulated the expression level of the CD8^+^ T cells activation marker 41BB^+^ (*P* = 0.023) and that LIPUS significantly enhanced the level of the anti-tumor cytokine IFN-γ secreted by CD8^+^ T cells (*P* = 0.001) (Fig. 6B). At the same time, LIPUS was found to significantly increase the levels of CXCL9 and CXCL10 (Fig. 6C), which are associated with the recruitment and activation of T cells and Natural Killer Cell (NK cells), at the early stage of irradiation. TUNEL staining additionally revealed a significant red apoptotic signal in the tumors of LIPUS mice (Supplementary Fig. 5). These results suggested that LIPUS significantly remodeled the tumor microenvironment, including immune cell infiltration and activation of CD8^+^ T cells, as well as significant changes in chemokines and cytokines, and may promote apoptosis in tumor tissues.

## Discussion

The spleen is an essential immune organ responsible for the maturation and function of T cells. The spleen contains a large number of DCs, macrophages and helper T (Th) cells [19], which facilitate the activation and function of cytotoxic T lymphocyte (CTL). In this study, we first demonstrated the ability of LIPUS to inhibit tumor development in a 4 T-1 mouse model of breast cancer by stimulating the spleen in order to activate the immune system and further reverse the immunosuppressive state in the tumor.

Our results revealed that when LIPUS irradiated normal mouse for 14 and/or 21 days, even at day 30 after inoculation with 4 T-1 tumors, LIPUS promoted the release of some critical cytokines such as IL-2, IL-7 and IFN-γ in the plasma of tumor-bearing mice. IL-2 belongs to Th1 cells, which mediate cellular immunity and are a critical component of T lymphocyte activation and differentiation [20]. IL-7 is intrinsically involved in T cell homeostasis as an essential cytokine that enhances the persistence of T cells in vivo [21]. Our previous study [22] found that IL-2 and IL-7 levels in the spleen were still elevated in middle-aged (12–14 months old) C57BL/6 mice after LIPUS underwater irradiation and after one month of follow-up. The present study additionally demonstrates the significant impact of LIPUS on the release of upgraded IL-2 and IL-7. However, it is also of interest to find that the activation effect of LIPUS on T cells during this process is not robustly significant and no significant infiltration of activated CD8^+^T cells is observed in the tumor microenvironment. However, LIPUS significantly increased the activation and proliferation of CD4^+^ and CD8^+^ T cells when tumors were already present in the mice. We speculate that this may be due to the limited response of normal mice to LIPUS immune activation when LIPUS is irradiated in such a manner. It appears that when tumor antigens are present in the body, the effects of LIPUS tip the balance of the immune state in favor of an anti-tumor response. Therefore, our study focused primarily on the significant enhancement of anti-tumor immune effects in the spleen of LIPUS-irradiated tumor-bearing mice.

CD4^+^ T cells have an essential role in inducing the generation and maintenance of anti-tumor immune responses [23]. Tregs are critical for maintaining immune homeostasis, not only suppressing antitumor responses but also leading to immune tolerance to tumors [24]. In this study, LIPUS was found to significantly downregulate Tregs in the spleen, improve immune tolerance in the spleen, and significantly induce activation of CD4^+^ T cells in the spleen. It also increased the proportion of Tfh cells in the spleen, and the accumulation of Tfh cells was shown in a recent report to be a major contributor to CD8^+^-dependent anti-tumor immunity and anti-PD-L1 efficacy [25]. Secondly, LIPUS significantly induced activation of CD8^+^ T cells and increased secretion of cytokines in the spleen, particularly significantly enhancing the production of the anti-tumor cytokine IFN-γ secreted by CD8^+^ T cells. IFN-γ elicits a potent antitumor immune response by inducing a Th1 response and CTL activation [26]. Previous studies have provided evidence supporting the potential use of ultrasound for immune system regulation. Zachs et al. [15]. used noninvasive ultrasound stimulation of the spleen to alter the expression of genes involved in cytoskeletal regulation, thereby affecting lymphocyte polarization or migration. We also confirmed by transcriptome sequencing that LIPUS exerts positive effects in the positive regulation of T cell proliferation and chemotaxis, T cell receptor V(D)J recombination, adaptive immune response, activation of T cells and positive regulation of macrophage chemotaxis. These combined effects activate immune function in the spleen and additionally induce an effective anti-tumor immune response.

Although LIPUS was not used to irradiate the primary tumor, we also examined lymphocyte infiltration in the tumor microenvironment. Our data showed that LIPUS-induced immune infiltration of CD45^+^ leukocytes and CD8^+^ T cells in tumors with a significant increase in the expression level of the CD8^+^ T cell activation marker 41BB^+^, which was shown to be one of the most prominent activation markers expressed on CD8^+^ T cells [27]. CD8^+^ T cells secreting IFN-γ were similarly upregulated, indicating an enhanced anti-tumor function of their infiltrating CD8^+^ T cells. LIPUS can increase the expression of CD4^+^ T cells in peripheral blood, but not CD8^+^ T cells (Fig. 2E–F), while in the spleen, LIPUS activates CD8^+^ T cells, but not CD4^+^ T cells (Fig. 2G). Cole et al. [28] find that spleen was a significant decrease in the frequency of CD4^+^ cells as compared to the peripheral blood and a significant increase in the frequency of CD8^+^ T cell, and higher frequency of CD4^+^ T cells than CD8^+^ T cells in peripheral blood. Consistent with the conclusions from Velásquez-Lopera et al. [29], at the same time, LIPUS was able to significantly increase the levels of chemokines CXCL9 and CXCL10, which are associated with T cells and NK cells recruitment and activation [30–32]. As the most influential immune activators of chemotactic CD8^+^ T cells homing, they significantly induced immune infiltration of the tumor during the early stages of LIPUS irradiation. The role of IL-10 in tumor development is controversial. The main role of IL-10 at the onset of tumor formation may be to stimulate NK cells and CTL-mediated killing of tumor cells. However, if tumor cells survive and rearrange, then IL-10 in the tumor microenvironment may act as a powerful tumor promoter [33]. Clearly, IL-10 acts as an immunosuppressive factor here, and its reduced levels further confirm that LIPUS may suppress its activity in the tumor microenvironment. The preexisting tumor-infiltrating lymphocytes are strong predictors of immunotherapeutic response [34]. All of these results suggest that LIPUS reverses the state of immunosuppression in tumors and that the effects of LIPUS on enhancing the immunotherapeutic response should not be overlooked.

Currently, the experiment is in its infancy and there are still several limitations. First, we observed differences in treatment outcomes among mice may be due to individual differences (Supplementary Fig. 6). Therefore, large size samples will play a key role in verifying these results in the next study. Second, we found that the immune-enhancing effects of LIPUS may be associated with CD8^+^ T cells, but details such as whether CD8^+^ T cells are the target cells for LIPUS to work are uncertain, and whether there is another target cell that is more sensitive to ultrasound is unknown. Third, in this study we only looked at the tumor microenvironment and were short to detect relevant changes in the immunity of tumor cells. Fourth, LIPUS are not focused; thus, the precise target of LIPUS irradiation on the spleen has not yet been accurately determined. ​We do not know whether the treatment would have worked better when we could have targeted more precise parts like the white pulp. All of the above requires us to conduct additional studies in the future to advance LIPUS as a potential cancer treatment.

In summary, we demonstrated that LIPUS irradiation of the spleen induces systemic immune activation and improves the immunosuppressive state of the tumor microenvironment, resulting in an effective anti-tumor immune response. This has significant implications in inhibiting tumor growth. LIPUS provides a noninvasive way to irradiate the spleen, thereby activating the immune system, providing a modern approach and an effective adjunct to immunotherapy in oncology.

### Supplementary Information

Below is the link to the electronic supplementary material.

Supplementary file1 (DOCX 8224 KB)

## Data Availability

All data generated or analyzed during this study are included in this published article.
