# Supplementary material for: Low-intensity pulsed ultrasound activated the anti-tumor immunity by irradiating the spleen of mice in 4 T-1 breast cancer
Source: Cancer Immunol Immunother. 2024 Feb 13;73(3):50. doi: 10.1007/s00262-023-03613-1 (PMC10864467; doi:10.1007/s00262-023-03613-1)
Supplement: Supplementary file 1 — Supplementary file1 (DOCX 8224 KB) [file 262_2023_3613_MOESM1_ESM.docx]

**Supplementary Materials**

**Low-intensity pulsed ultrasound activated the anti-tumor immunity by irradiating the spleen of mice 4T-1 breast cancer**

Yi Xia^1^, Meijie Yang^1,2^, Xinfang Xiao^1^, Wentao Tang^1^, Juan Deng^1^, Liu Wu^1^, Haopeng Xu^1^, Yilin Tang^1^, Wenzhi Chen^1^, Yan Wang^1*^

^1^State Key Laboratory of Ultrasound in Medicine and Engineering, College of Biomedical Engineering, Chongqing Key Laboratory of Biomedical Engineering, Chongqing Medical University, Chongqing, China

^2^ College of Medical Informatics, Chongqing Medical University, Chongqing, China

***Corresponding author:**

**Yan Wang**

State Key Laboratory of Ultrasound in Medicine and Engineering, Chongqing Medical University, 1 Yixueyuan Rd, Yuzhong District, Chongqing, China.

Tel: +86 13883274124

Email: [wangyancq@cqmu.edu.cn](mailto:wangyancq@cqmu.edu.cn)


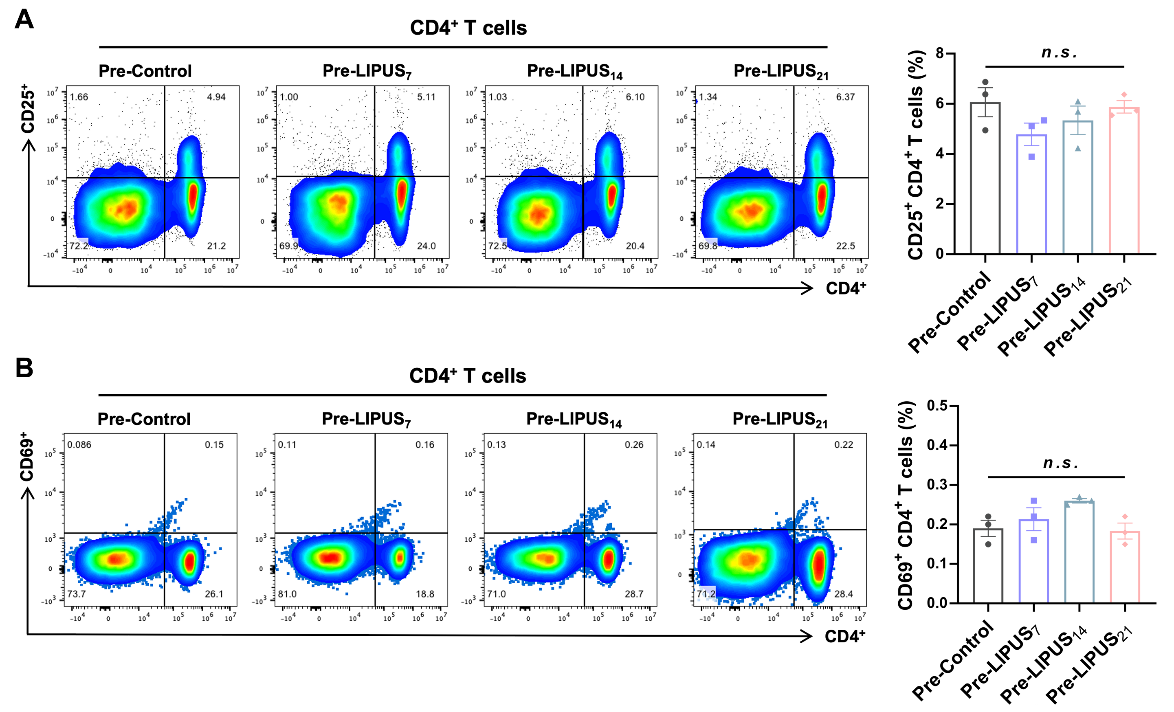


**Supplementary Fig. 1. LIPUS had no obvious effect on the activation of CD4^+^ T cells and Tregs in the spleen of normal mice.** (A) Tregs in the spleen of tumor-bearing mice on day 30 (n = 3 per group, CD4^+^ T / lymphocyte). (B) Expression of CD69^+^ gated on CD4^+^ T cells in the spleen (n = 3 per group, CD4^+^ T / lymphocyte). Data are shown as mean ± SEM. *N.s.* no statistical significance.


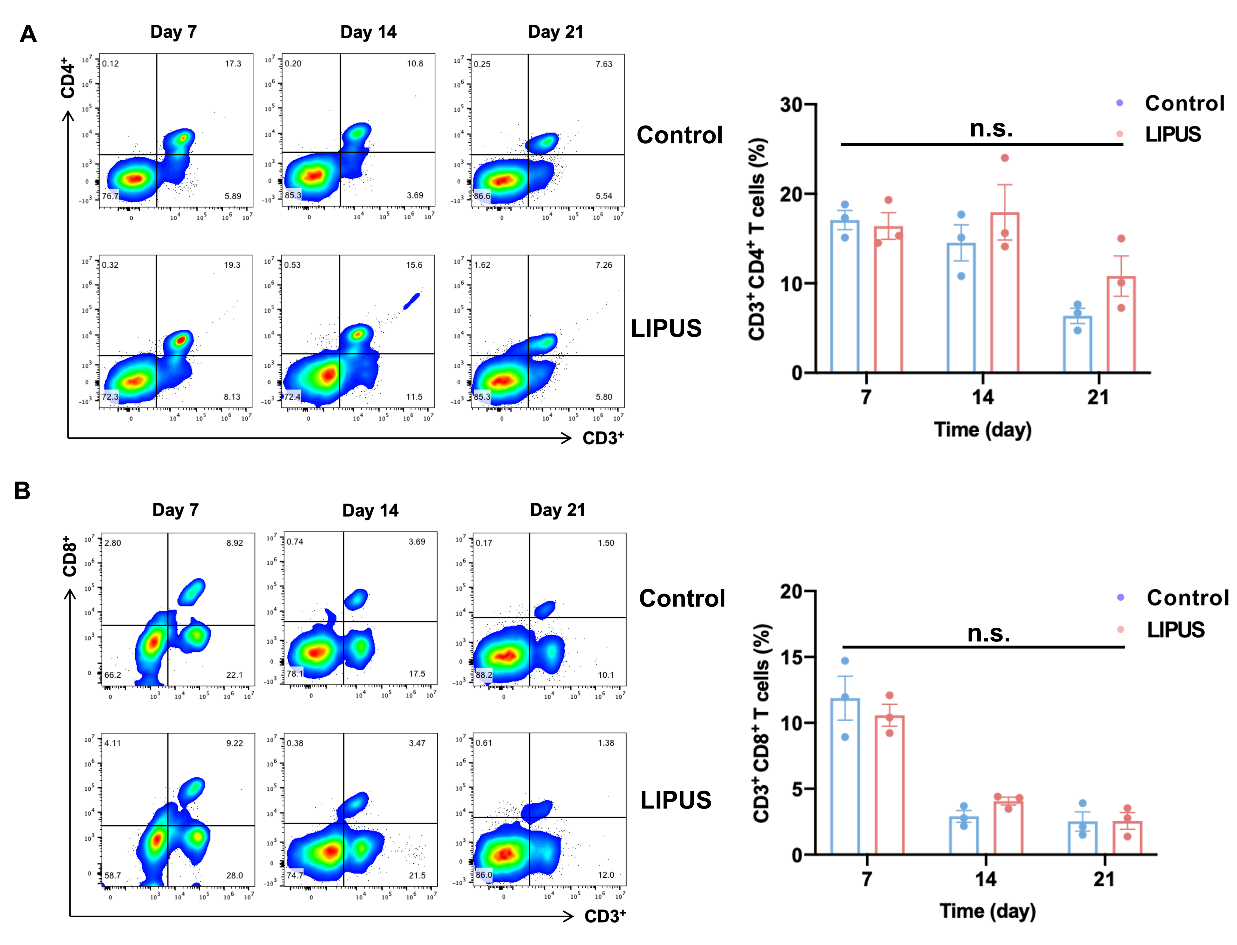


**Supplementary Fig. 2. LIPUS had no significant effect on adaptive immune cells in peripheral blood of tumor-bearing mice.** (A) Expression of CD3^+^CD4^+^ T cells in peripheral blood of tumor-bearing mice (n = 3 per group, CD4^+^ T / lymphocyte). (B) Expression of CD3^+^CD8^+^ T cells in the peripheral blood (n = 3 per group, CD4^+^ T / lymphocyte). Data are shown as mean ± SEM. *N.s.* no statistical significance.


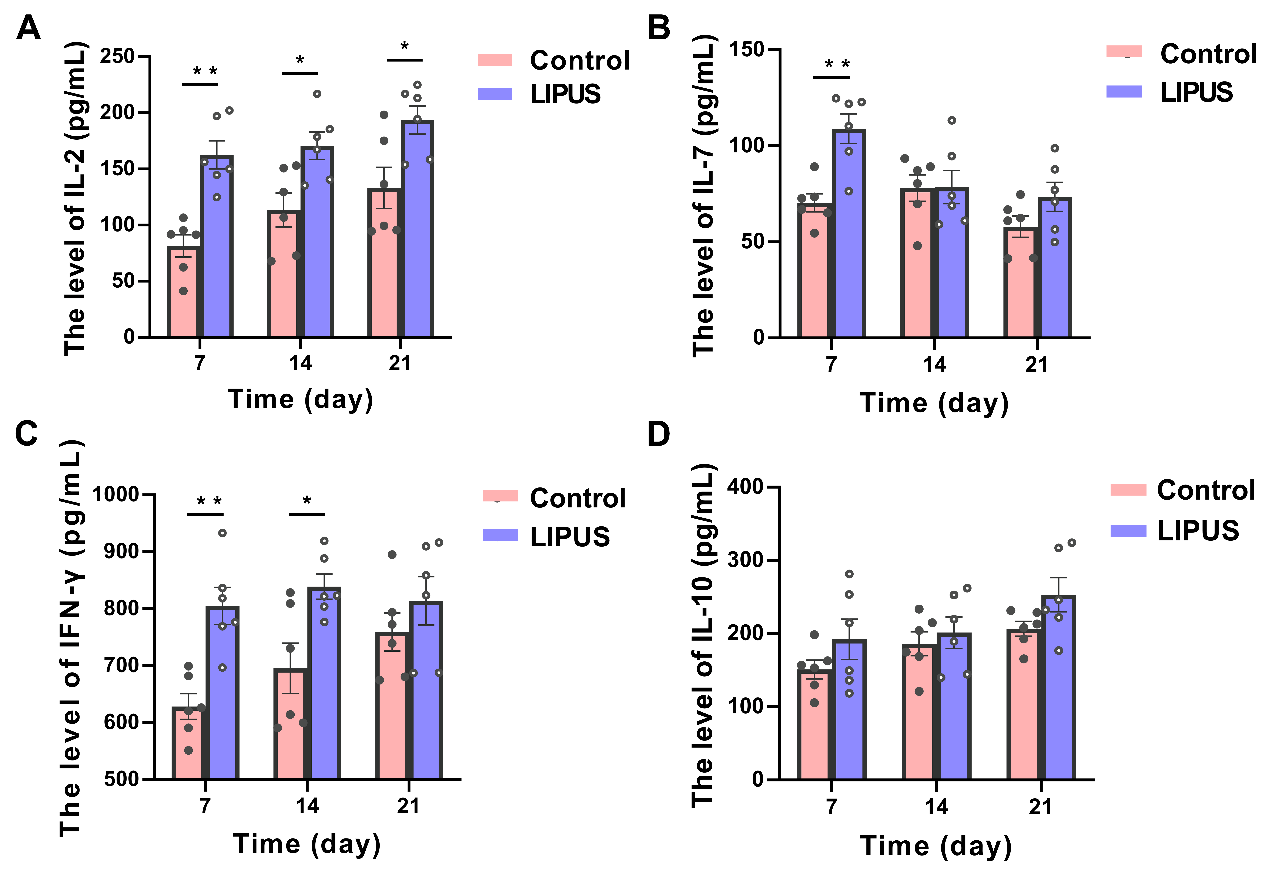


**Supplementary Fig. 3. LIPUS induced dynamic changes in cytokines in the serum of tumor-bearing mice.** The levels of (A) IL-2, (B) IL-7, (C) IFN-γ and (D) IL-10 in the serum of tumor-bearing mice (n = 6 per group). Data are shown as mean ± SEM. **P* < 0.05, ***P* < 0.01.

**
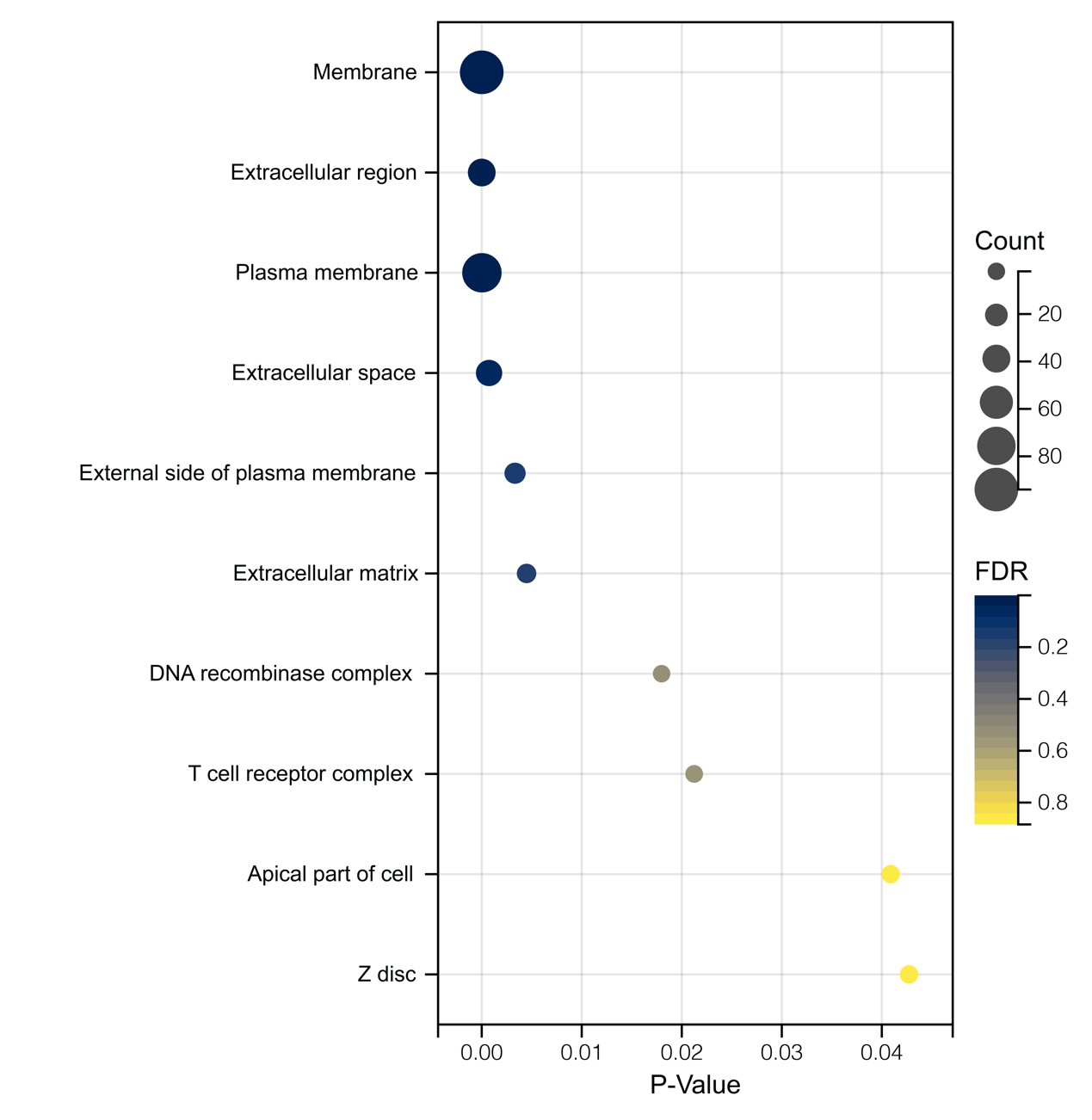
**

**Supplementary Fig. 4. Scatter plot for GO in terms of cellular localization (CC) enrichment results in the upregulated genes.** The dot size indicates the number of DEGs contained in the GO terms, and the dot depth indicates the extent of rich factor enrichment.


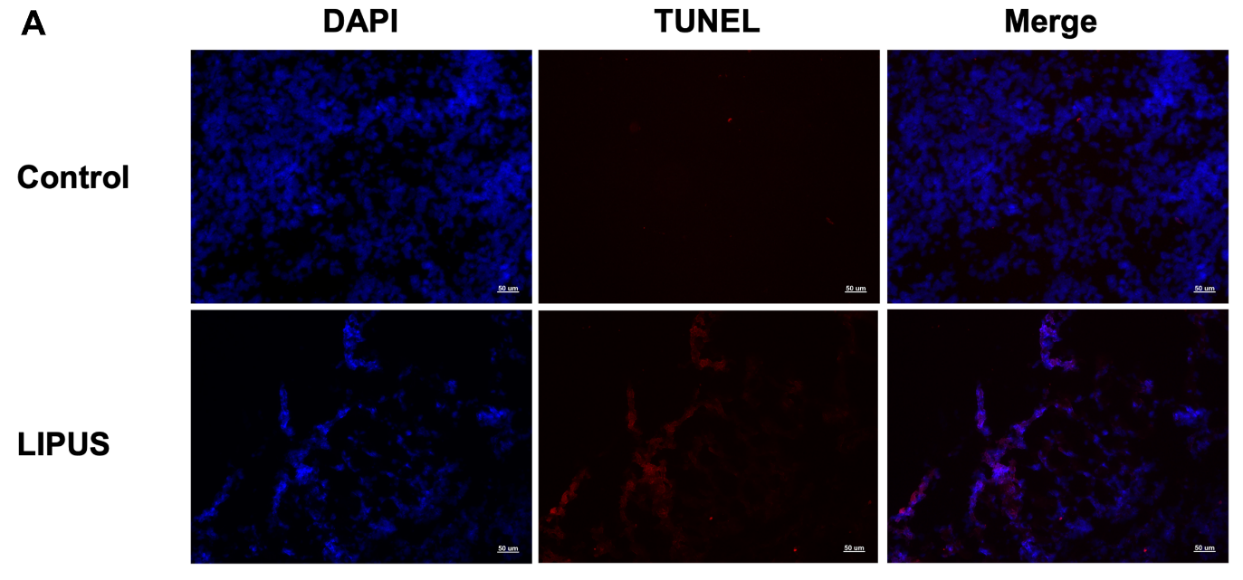


**Supplementary Fig. 5. TUNEL staining revealed a significant red apoptotic signal in the tumors of LIPUS mice.** TUNEL assay for tumor apoptosis. Nuclei were stained with DAPI and showed blue fluorescence, apoptotic cells showed red fluorescence (100×).


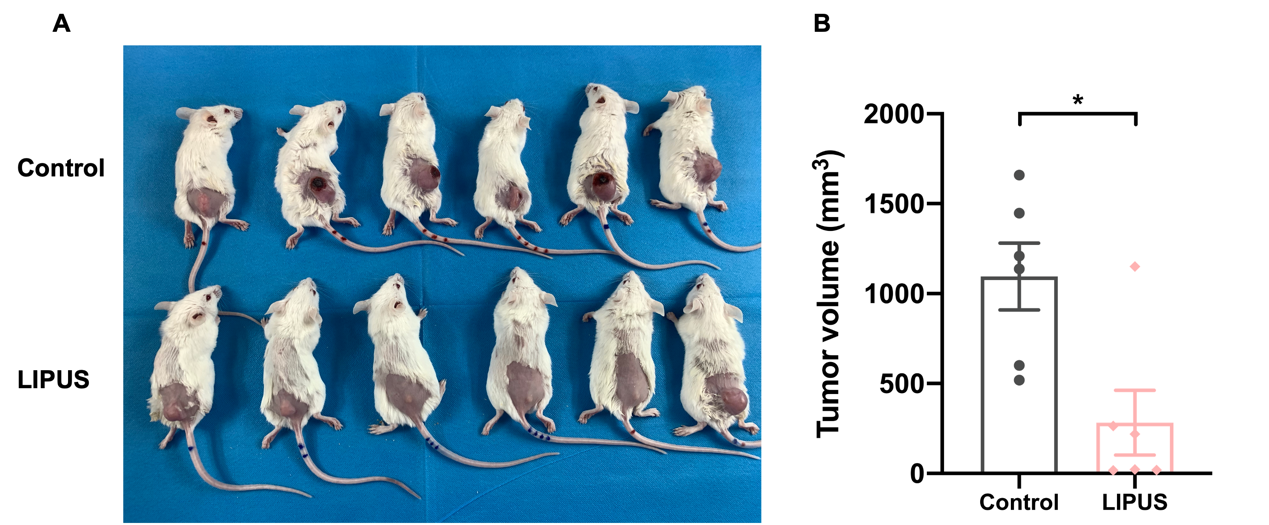


**Supplementary Fig. 6. Some variability in the treatment results of LIPUS across mice.** (A) Picture of tumor-bearing mice irradiated with LIPUS for 21 days in the spleen. (B) Tumor volume (n = 6 per group). Data are shown as mean ± SEM. **P* < 0.05.

**Flow cytometry (FCM)**

Peripheral blood, spleen and tumor are taken, with the spleen and tumor being cut and ground, and both eventually made into single cell suspension (1×10^7^/mL). Peripheral blood, spleens and tumors are labelled using appropriate antibodies according to the manufacturer's instructions. Cell surface antigen staining was first performed. For intracellular staining, cells were first stimulated with Cell Activation Cocktail (with Brefeldin A) at 37°C for 6 h. Cells were then fixed in Cyto-Fast™ Fix Buffer in the dark for 20 min at room temperature. Then these cells were resuspended in Cyto-Fast™ Perm Wash Solution and stained for IFN-γ antibody. For intranuclear staining, cells were permeabilized and fixed using a True-Nuclear™ Transcription Factor Buffer Set followed by FOXP3 antibody staining. Dead cells were excluded by Zombie NIR™ Fixable Viability Kit, and adherent cells were excluded by FSC/A and FSC/H gating analysis. Cells were analyzed using CytoFLEX flow cytometry (Beckman Counter, USA). Data were analyzed by FlowJo version 10.8.1 (Ashland, OR, USA).

Antibodies to Brilliant Violet 510™ anti-mouse CD3 antibody (Clone: 17A2, Cat. 100233), PerCP/Cyanine5.5 anti-mouse CD4 antibody (Clone: GK1.5, Cat. 100433), FITC anti-mouse CD8a Antibody (Clone: 53-6.7, Cat. 100705), APC anti-mouse CD25 antibody (Clone: PC61, Cat. 102011), PE anti-mouse CD137 antibody (Clone: 17B5, Cat. 106105), Brilliant Violet 605™ anti-mouse CD69 antibody (Clone: H1.2F3, Cat. 104529), Brilliant Violet 421™ anti-mouse CD45 antibody (Clone: 30-F11, Cat. 103133), APC/Cyanine7 anti-mouse CD185 (CXCR5) Antibody (Clone: L138D7, Cat. 145525), PE anti-mouse CD279 (PD-1) Antibody (Clone: 29F.1A12, Cat. 135205), Brilliant Violet 421™ anti-mouse IFN-γ Antibody (Clone: CMG1.2, Cat. 505829), Alexa Fluor® 647 anti-mouse FOXP3 Antibody (Clone: MF-14, Cat. 126407) and TruStain FcX™ PLUS (anti-mouse CD16/32, Cat. 156603) were purchased from Biolegend.
